# Estimating the Impact of Spatial Social Polarization on Dementia Disparities

**DOI:** 10.1093/geroni/igaf122.544

**Published:** 2025-12-31

**Authors:** Hoda Abdel Magid, Mengya Xu, Wei-Lin Wang, Evelyn Gonzalez, Julie Zissimopoulos

**Affiliations:** University of Southern California, Los Angeles, California, United States; University of Southern California, Los Angeles, California, United States; University of Southern California, Los Angeles, California, United States; University of Southern California, Los Angeles, California, United States; University of Southern California, Los Angeles, California, United States

## Abstract

Using a 20% sample of Medicare claims data (2010–2019, N > 4 million), we examined the association between spatial social polarization (SSP) and Alzheimer’s Disease and Related Dementias (ADRD) prevalence and incidence. SSP was measured using the Index of Concentration at the Extremes (ICE) across five domains: race, income, education, language proficiency, and homeownership. Logistic regression models were used to estimate the association between neighborhood SSP and incident ADRD, adjusting for age, race, socioeconomic status, and comorbidities. Significant racial disparities were observed in ADRD prevalence and incidence in 2019, with rates notably higher among Black and Hispanic populations at 8% and 3.5%, respectively. Despite a yearly decrease in incidence rates, significant associations were found between higher risks in ADRD and neighborhood-level SSP in race, income, and education, though the association with SSP in primarily spoken language was not significant. These findings suggest that neighborhood SSP is associated with ADRD prevalence and incidence, with stronger associations observed for race, income, and education domains. Further investigation into the role of spatial social polarization in ADRD disparities may help identify potential intervention targets for reducing neighborhood-driven inequities in cognitive health.

